# Mass spectrometry dataset of LC-MS lipidomics analysis of *Xenopus laevis* optic nerve

**DOI:** 10.1016/j.dib.2023.109313

**Published:** 2023-06-14

**Authors:** Emily Neag, Isabella Moceri, Faith Harvey, Ava J. Udvadia, Sanjoy K. Bhattacharya, Fiona L. Watson

**Affiliations:** a Bascom Palmer Eye Institute, Miller School of Medicine at University of Miami, Miami, FL, 33136, USA; b Miami Integrative Metabolomics Research Center, Miami, FL, 33136, USA; c College of Osteopathic Medicine, Michigan State University, East Lansing, 48824, MI, USA; d Department of Biology, Appalachian State University, Boone, NC 28608; e Department of Biology, Washington and Lee University, Lexington, VA, 24450, USA

**Keywords:** Optic nerve crush injury, Regeneration, Lipidomics, Frog

## Abstract

CNS injuries of the anuran amphibian, *Xenopus laevis*, are uniquely suited for studying the molecular compositions of neuronal regeneration of retinal ganglion cells (RGC) due to a functional recovery of optic axons disparate to adult mammalian analogues. RGCs and their optic nerve axons undergo irreversible neurodegeneration in glaucoma and associated optic neuropathies, resulting in blindness in mammals. Conversely, *Xenopus* demonstrates RGC lifetime-spanning regenerative capabilities after optic nerve crush [1], inciting opportunities to compare de novo regeneration and develop efficient pharmaceutical approaches for vision restoration. Studies revealing lipidome alterations during optic nerve regeneration are sparse and could serve as a solid foundation for these underlying molecular changes. We profile the lipid changes in a transgenic line of 1 year old *Xenopus laevis Tg*(*islet2b:gfp*) frogs that were either left untreated (naïve) or had a monocular surgery of either a left optic crush injury (crush) or sham surgery (sham). Matching controls of uninjured right optic nerves were also collected (control). *Tg*(*islet2b:gfp*) frogs were allowed to recover for 7,12,18, and 27 days post optic nerve crush. Following euthanasia, the optic nerves were collected for lipidomic analysis. A modified Bligh and Dyer method [2] was used for lipid extraction, followed by untargeted mass spectrometry lipid profiling with a Q Exactive Orbitrap Mass Spectrometer coupled with a Vanquish Horizon Binary UHPLC LC-MS system (LC MS-MS). The raw scans were analyzed and quantified with LipidSearch 5.0 and the statistical analysis was conducted through Metaboanalyst 5.0. This data is available at Metabolomics Workbench, study ID [ST002414].

**Specifications Table**

**Table:** 

Subject	Ophthalmology
Specific subject area	Lipids in neuronal regeneration
Type of data	Chart Graph Figure Chromatograms Spectra
How data were acquired	High-Performance Liquid Chromatography, Q Exactive Orbitrap Mass Spectrometer
Data format	Raw Analyzed Filtered
Description of data collection	Optic nerve samples consist of pools of 3 animals per replicate. Post-metamorphic transgenic *Tg(Islet2b:GFP)* albino *Xenopus laevis* frogs, 6 - 8.0 cm in length, were either left untreated (naïve) or underwent a monocular surgery (operated; Fig. 1). Operated individuals were anesthetized with 0.05% ethyl 3-aminobenzoate methanesulfonate (Sigma, USA) and received either a crush injury to the left optic nerve (CX_L-ON crush; Fig. 1) and no treatment to the contralateral control right optic nerve (CTL_R-ON; Fig. 1). One group received a mock or sham surgery to the left eye (SHAM_L-ON; Fig 1) and no treatment to the contralateral control right eye (CTLSh_R-ON; Fig. 1). Optic nerves were collected, and lipid extraction was performed with the Bligh and Dyer method. The samples were analyzed for an untargeted lipid profile using LC MS-MS.
Data source location	Bascom Palmer Eye Institute, Miller School of Medicine at University of Miami, Miami, FL 33136, USA
Data accessibility	This study is available at the NIH Common Fund's National Metabolomics Data Repository (NMDR) website, the Metabolomics Workbench, https://www.metabolomicsworkbench.org where it has been assigned Study ID **ST002414**. The data can be accessed directly via its Project DOI: doi: 10.21228/M8TM6T. This work is partly supported by NIH grant **U01EY027257, P30EY014801**.

## Value of the Data

• The data provides insights into the lipid changes that occur in *Xenopus laevis* after traumatic optic nerve injury and subsequent axonal regeneration.
• This data can be used to further study the lipidomic changes associated with optic nerve regeneration.
• While the adult frog regenerates optic nerve retinal ganglion cells post crush, humans do not have this innate regenerative capability. This distinction leads to investigation of changes in post-injury responses between species and can be used to inform regenerative therapies.

## 1 Objective

The axon outer boundary of the optic nerves is comprised of lipids. Injured axons require large amounts of lipids for axon regeneration to occur. The mass spectrometric analysis of the lipidome in animals undergoing axon regeneration versus control will help generate data that can identify specific lipids associated with regenerating retinal ganglion cell axons. Identifying comprehensive changes across the lipid classes is the objective of experiments carried out to generate the dataset.

## 2 Data Description

**Fig. 1:**
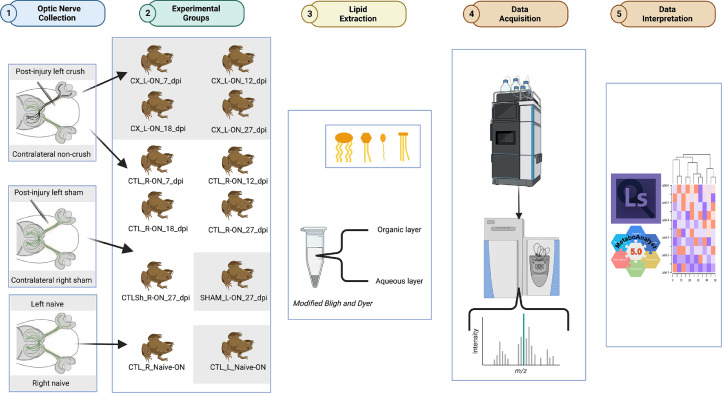
**Graphical abstract illustrating the experimental workflow.** Schematic depicting from optic nerve sample collection to data acquisition and interpretation. Created with BioRender.com.

**Fig. 2:**
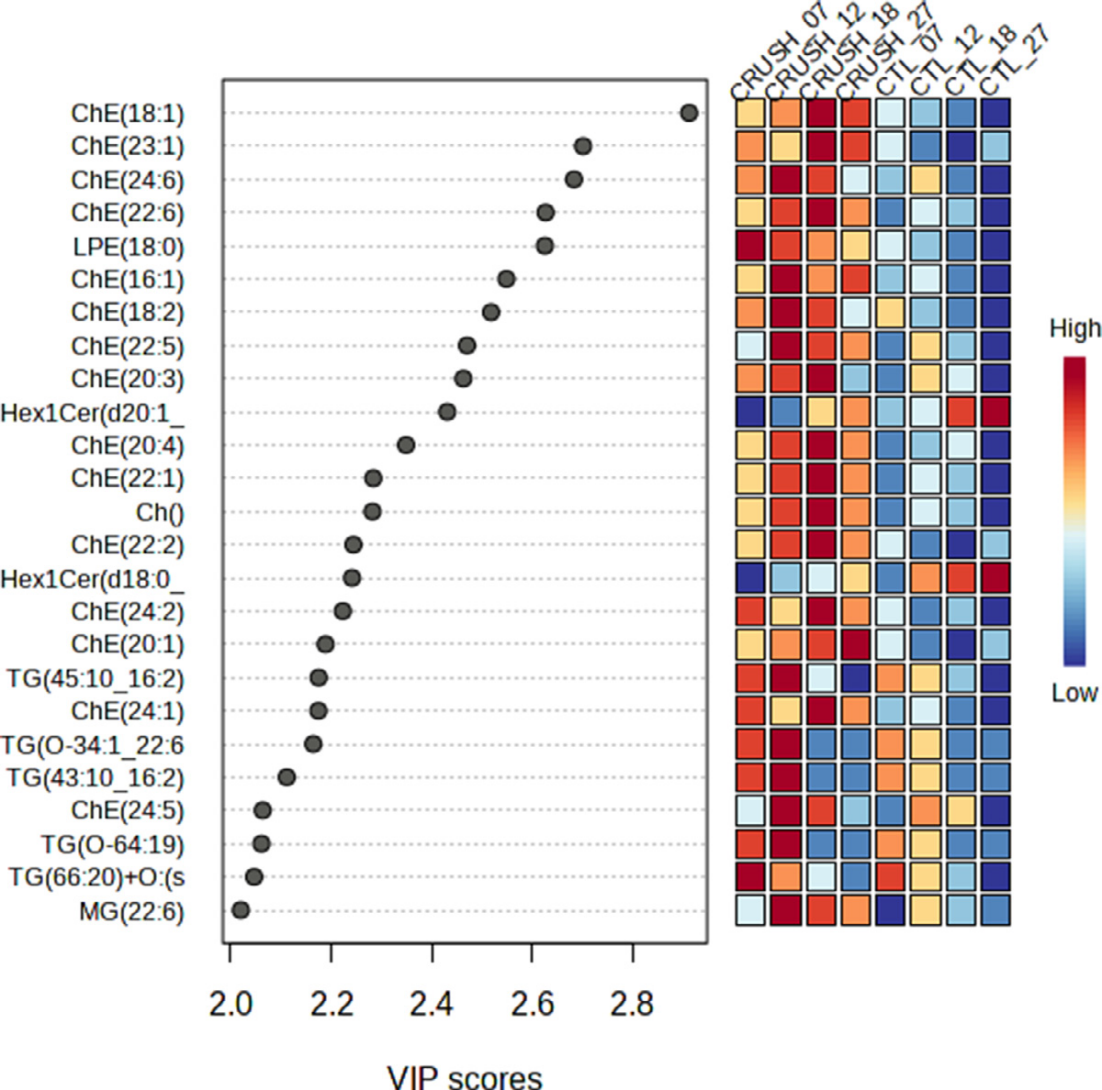
**Top twenty-five important lipid features identified by partial least square-discriminant variable importance in projection (VIP) scores.** VIP scores in regeneration groups (7-, 12-, 18-, and 27-days post injury) compared to sham controls. The colored boxes on the right indicate the relative concentrations of the corresponding lipid species.

**Fig. 3:**
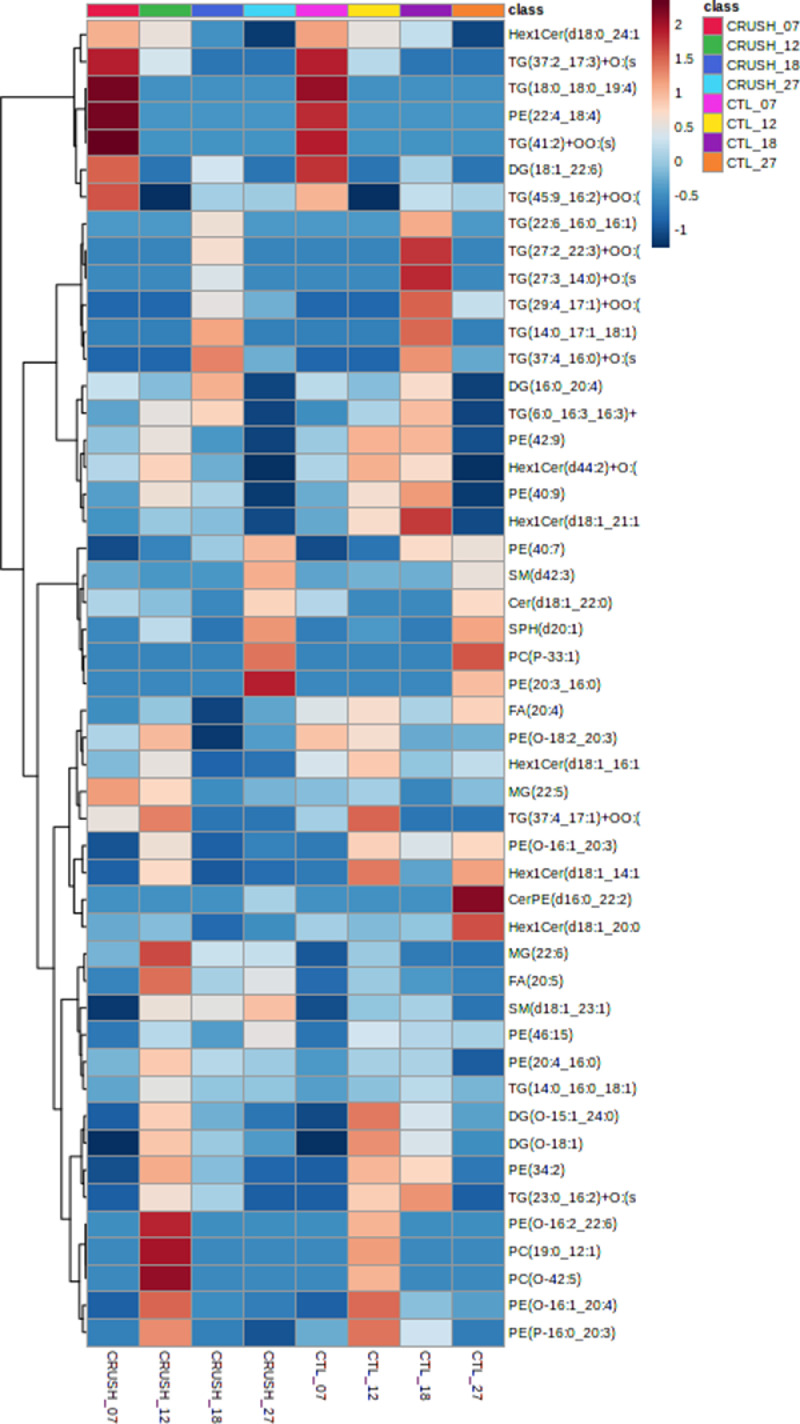
**Heatmap showing lipid abundances of the top fifty lipid species.** Heatmaps showing post optic nerve crush (7-, 12-, 18-, and 27-days post injury) compared to sham controls. Euclidean distances and ward clustering were used.

Lipidomics analysis was performed on crushed optic nerves from adult frogs compared to uncrushed optic nerves from control frogs. To evaluate the regenerative capacity of RGC axons following an optic nerve crush injury, the nerves were collected at 7-, 12-, 18- and 27-days post injury. Lipids were extracted using a Bligh and Dyer method and spiked with internal standard before injection. Extraction blanks were created throughout the extraction process to control for contamination. Each sample was separated using a Vanquish Horizon Binary UHPLC LC-MS and run in both positive and negative mode on a Q Exactive Orbitrap mass spectrometer. The raw data was processed and analyzed using LipidSearch 5.0. All scans per sample were aligned in LipidSearch 5.0 for processing and analysis. Additional analysis was performed on Metaboanalyst 5.0. Quantification of lipids for each class was performed using the EquiSPLASH Lipidomix Quantitative Mass Spec internal standards. The Statistical analysis was performed using Metaboanalyst 5.0 (Fig. 1). Multivariate statistical analysis was performed. We have performed principal component analysis (PCA). We also performed partial least square-discriminant analysis (PLS-DA) and hierarchical clustering heatmaps (Figs. 2 and 3).

## 3 Experimental Design, Materials and Methods

### 3.1 Animals

**Fig. 4:**
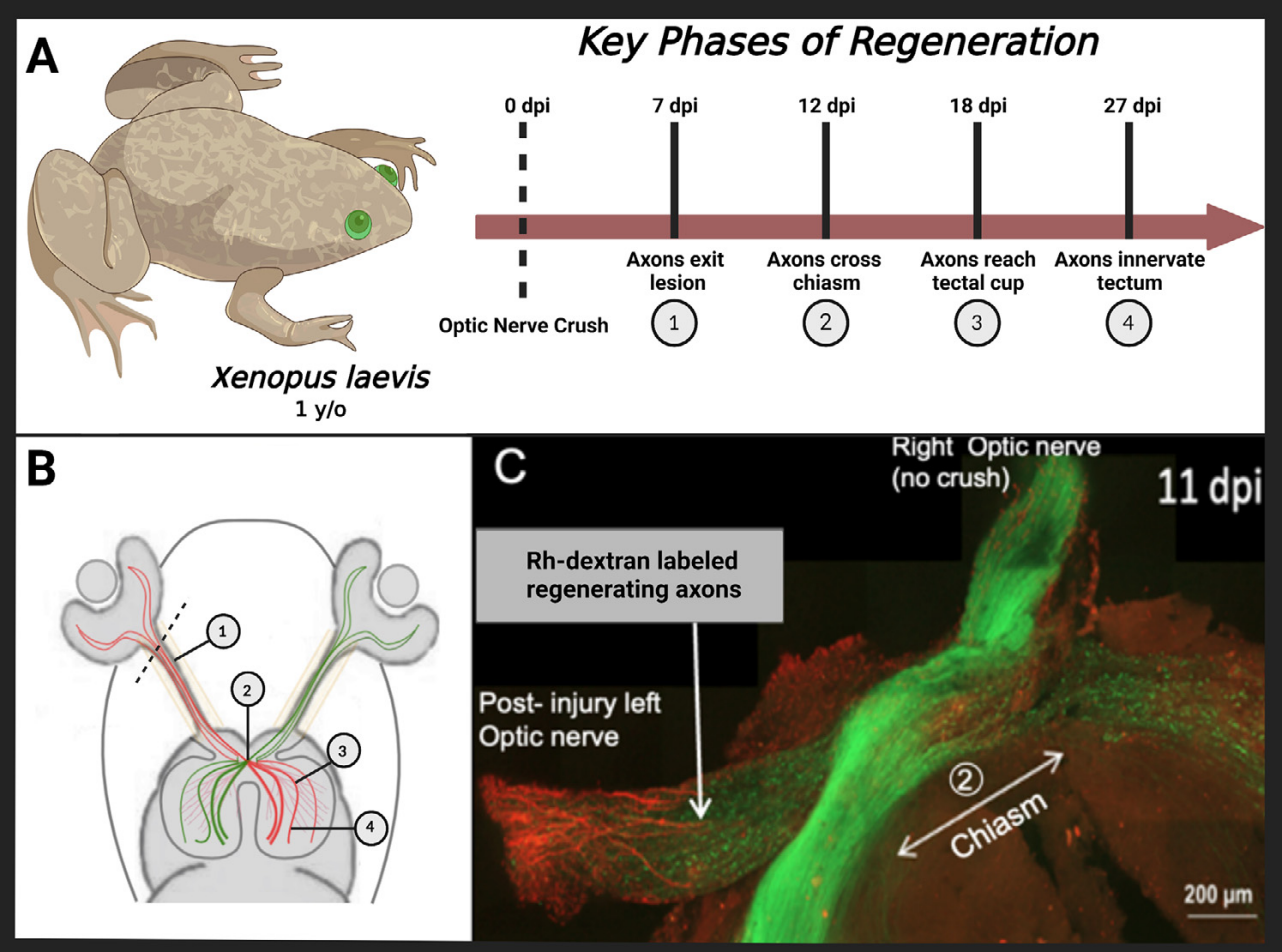
**Graphical illustration showing the regeneration timeline for axonal regrowth.** The four key phases of regeneration in one-year old adult frogs occurs at 7, 12, 18, and 27 – days post injury (dpi) (A, B). Representative image shows stacked confocal image from adult *Xenopus* frogs [*Tg(Islet2b:GFP*)] expressing GFP (green) and post-injury rhodamine dextran labeled axons (red) (C). At 11 dpi, RGC axon regrowth approaches the optic chiasm (double arrow) and has reached the chiasm at 12dpi (not shown). A, B created with BioRender.com.

Experiments were carried out using F1 progeny from a single albino female transgenic frog line *Tg*(*Islet2b:GFP*) mated with a wildtype albino male frog described previously [3]. These progeny were grown to post-metamorphic stage (> 1 year) under 12L:12D photoperiod at 22°C. *Xenopus laevis* frogs, 6-8 cm in length, were either left untreated (naïve) or underwent a monocular surgery (operated). Operated individuals were anesthetized with 0.05% ethyl 3-aminobenzoate methanesulfonate (Sigma, USA) and received either a sham surgery (L-ON sham) or a crush injury (L-ONX crush) to the left optic optic nerve, and no surgical treatment to the contralateral right optic nerve (R-ON control). The optic nerve was accessed dorsally by cutting the conjunctiva above the eye and tipping the eye forward [4]. Surgeries were conducted under the epi-fluorescent microscope to clearly identify the optic nerve using fluorescence. We crush the optic nerve for 5 seconds using forceps. While this technique briefly stops blood flow to the retina, the blood flow recovers quickly and no long-term tissue damage is visible*.* RGC axons of surgically manipulated *Tg*(*islet2b:GFP*) frogs were allowed to recover for 7, 12, 18, and 27 days post injury (dpi). These post injury time points were shown to align with the four key phases of axon regeneration (Fig. 4A and B; unpublished). The nerve segments collected were from the back of the eye orbit and extended to but did not include the optic chiasm. Optic nerve samples from frogs were collected quickly and immersed in liquid nitrogen, placed on dry ice and stored at -80°C for later lipid extraction and processing. Each sample contained optic nerves from three individual frogs that were pooled. The sex of the frogs is not known to affect optic nerve regeneration. All animal experiments were carried out using procedures approved by the Washington and Lee University's Institutional Animal Care and Use Committee (IACUC).

### 3.2 Optic nerve regeneration visualization

RGC axons exit the crush injury site at 7 dpi, cross the optic chiasm at 12 dpi (see axons extending towards the optic chiasm at 11dpi; Fig. 4C), reach the tectal cup at 18 dpi and establish synaptic connections in the different layers of the tectum by 27 dpi (Fig. 4A, B; unpublished). Optic nerves receiving a crush injury are easily identified by the punctate GFP expression pattern of the distal degenerating RGC axons (Fig. 4C). To visualize regrowth of RGC axons following an optic nerve crush injury, two days prior to harvesting tissue, 2 µl of 10 mg/ml of rhodamine dextran (3,000 MW; In vitrogen) was injected intravitreally followed by two 30 mSec pulses (50 mV) using an electroporator (Caltech). Eyes, optic nerves and tectal tissues were then dissected in 1 x phosphate buffered saline (1 X PBS), placed under a coverslip overnight in 4% paraformaldehyde, then washed in 1 x PBS and mounted under a 1.5 cover glass using fluorumount-G with DAPI (Southern Biotech). Images were acquired using a Zeiss LSM 900 confocal microscope with Plan-Apochromat 20X/0.8 M27 objective. Overlapping tiles of Z-stacked images were acquired in the green and red channels. Specimens encompassed the optic nerves, chiasm, optic tracts and tecta. Ortho-projections and stitching of images were conducted using Zeiss Zen Blue software.

### 3.3 Lipid extraction

Lipids were extracted from the optic nerve tissue with a Bligh and Dyer method. The organic phase containing the lipids was removed after centrifugation and dried down with a vacuum centrifuge. The lipids were flushed with argon gas to prevent oxidation and stored at -80°C prior to analysis.

### 3.4 High performance liquid chromatography and mass spectrometry

Dried lipid samples were reconstituted in 49µl of isopropanol:acetonitrile 1:1 (v/v) and 1µl of EquiSPLASH™ LIPIDOMIX® Quantitative Internal Standard (330731) and sonicated for 15 minutes for total solubilization. Samples were split into two separate vials containing 25µl each, one for positive mode and one for negative mode. Reversed phase chromatographic separation was performed on Vanquish Horizon UHPLC system (Thermo) using an Accucore Vanuqish C18+ UHPLC Column. An injection volume of 5µl was used and the flow rate was 260 µl/min. Mobile phase A was 50% acetonitrile, 50% water, 5mM ammonium formate, and 0.1% formic acid. Mobile phase B was 88% isopropanol, 10% acetonitrile, 2% water, 5mM ammonium formate and 0.1% formic acid.

Ionization and detection were performed with a heated electrospray ionization (HESI) source coupled to a Q Exactive mass spectrometer. Data was collected in both positive and negative modes for each sample. Spray voltage was 4.0kV in positive mode and 2.50 kV in negative mode. For positive and negative mode, sheath gas flow rate was 35, auxiliary gas flow rate was 15, and sweep gas was 0. Capillary temperature was 325 and S-lens RF level was 70. The full scan range was 250 to 1200 m/z, resolution was 70,000 and microscans was 1. AGC target was 1e6 and maximum inject time was 100ms. In dd-MS2, the resolution was 17,500, AGC target was 1e5, loop count was 10, and isolation window was 1.0m/z. NCE was set to 20, 30, 40, intensity threshold was 2.0e4, and dynamic exclusion was 8.0s.

### 3.5 Raw scan processing and bioinformatics

The raw scans were processed using LipidSearch 5.0. LC-MS Product Search was performed with a Precursor Tolerance of 5.0ppm, Product Tolerance of 8.0ppm, and Product Threshold of 1.0ppm. Lipid species identified in positive and negative mode were aligned for each sample. LC-MS Alignment was performed using a Retention Tolerance of 0.05min, Retention Correction Tolerance of 0.5min, Signal to Noise Threshold of 3.0, Intensity Ratio Threshold of 1.5, and Valid Peak Rate Threshold of 0.5. Quantitation was carried out using the deuterated lipid standard for each lipid class. Data was further analyzed in Metaboanalyst 5.0. Missing values were excluded from analysis and data was filtered using Interquantile Range (IQR), and transformed using log transformation (base 10).

## Ethics Statements

All animal experiments were carried out using procedures approved by the Washington and Lee University's IACUC in compliance with the National Institutes of Health guide for the care and use of laboratory animals (NIH Publications No. 8023, revised 1978). The animals in this study were 90% female and 10% male with the same ratio of female:male animals per timepoint. The sex of the frogs is not known to affect optic nerve regeneration*.*

## CRediT Author Statement

**Emily Neag:** Writing - Original Draft, Investigation, Data Curation, Visualization; **Isabella Mocer:**  Investigation; **Faith Harvey:** Writing - Review & Editing; **Ava J. Udvadia:** Writing - Review & Editing; **Sanjoy K. Bhattacharya:** Conceptualization, Methodology, Supervision, Funding acquisition, Writing - Review & Editing, Project administration; **Fiona L. Watson:** Methodology, Investigation, Supervision, Funding acquisition, Writing - Review & Editing.

## Declaration of Competing Interest

The authors declare no competing financial or personal relationships that may have influenced the work presented in this article.

## Data Availability

Mass spectrometry dataset of LC-MS Lipidomics Analysis of Xenopus Laevis Optic Nerve (Original data) (Metabolomics Workbench).

## Appendix Supplementary materials

Image, application 1

Image, application 2
